# Tumor microenvironment in oral squamous cell carcinoma

**DOI:** 10.3389/fimmu.2024.1485174

**Published:** 2024-12-18

**Authors:** Chenxi Li, Xiaodan Dong, Bo Li

**Affiliations:** Department of Oral Anatomy and Physiology, Jilin Provincial Key Laboratory of Oral Biomedical Engineering, Hospital of Stomatology, Jilin University, Changchun, China

**Keywords:** oral squamous cell carcinoma, tumor microenvironment, tumor-associated macrophages, interaction, cellular network

## Abstract

Oral squamous cell carcinoma (OSCC) is a highly aggressive and malignant tumor of oral cavity with a poor prognosis and high mortality due to the limitations of existing therapies. The significant role of tumor microenvironment (TME) in the initiation, development, and progression of OSCC has been widely recognized. Various cells in TME, including tumor-associated macrophages (TAMs), cancer-associated fibroblasts (CAFs), T lymphocytes, tumor-associated neutrophils (TANs), myeloid-derived suppressor cells (MDSCs) and dendritic cells (DCs), form a complicated and important cellular network to modulate OSCC proliferation, invasion, migration, and angiogenesis by secreting RNAs, proteins, cytokines, and metabolites. Understanding the interactions among cells in TME provides the foundation for advanced clinical diagnosis and therapies. This review summarizes the current literature that describes the role of various cellular components and other TME factors in the progression of OSCC, hoping to provide new ideas for the novel OSCC treatment strategies targeting the complicated cellular network and factors that mediate the interactive loops among cells in TME.

## 1 Introduction

**Figure 1:**
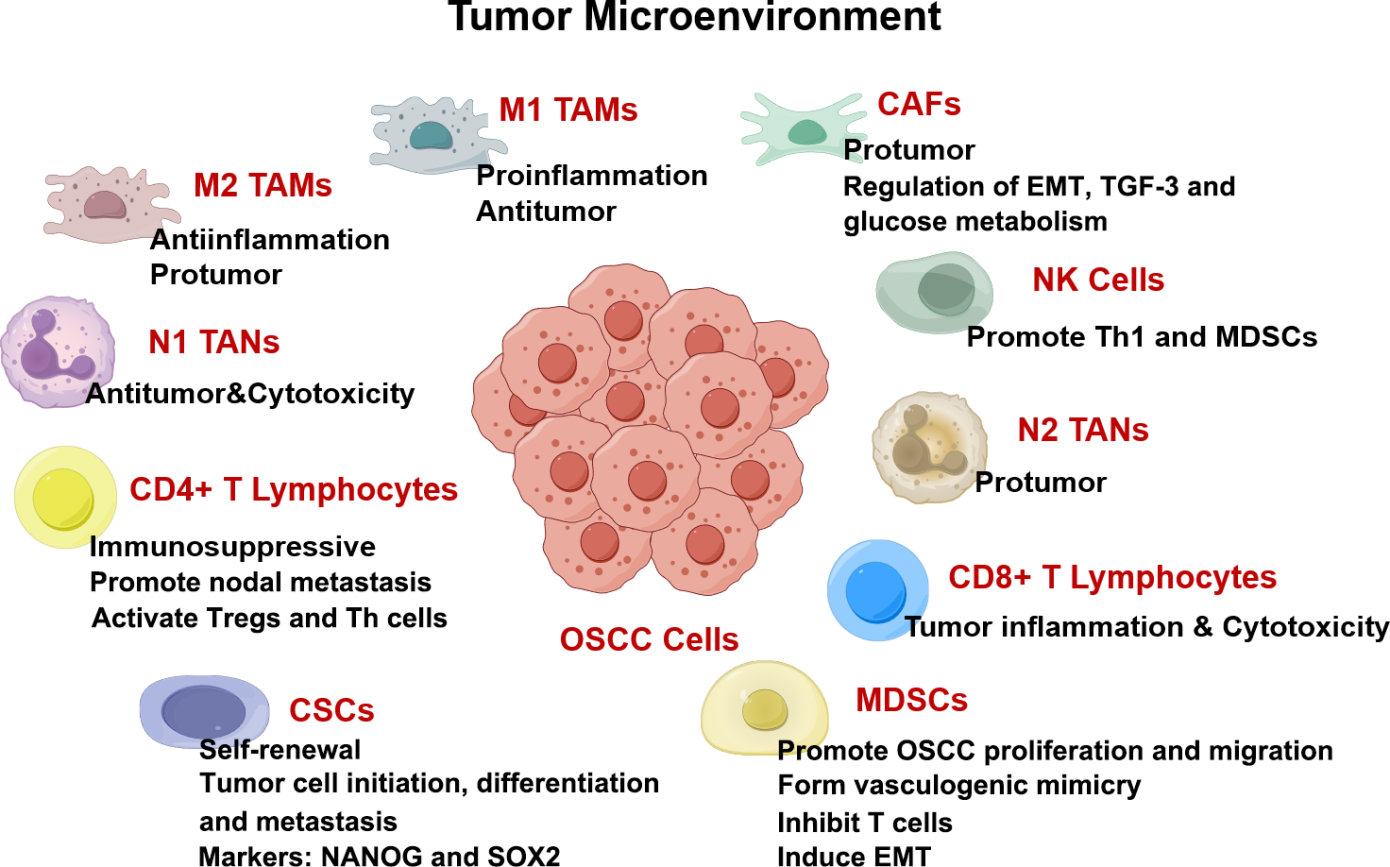
TME in OSCC drawn by Figdraw. Components in TME including cancer-associated fibroblasts (CAFs), myeloid-derived suppressor cells (MDSCs), T lymphocytes, tumor-associated neutrophils (TANs), tumor-associated macrophages (TAMs), and cytokines, are all intricately interconnected with the angiogenesis, proliferation, invasion, and migration of OSCC.

Oral squamous cell carcinoma (OSCC) is a highly aggressive and malignant tumor of oral cavity. The number of new cases and deaths in 2020 is 377713 and 117757 respectively (1). Smoking, alcohol abuse, betel nut chewing, and genetic factors are strongly associated with OSCC occurrence (2). The incidence of OSCC is also regional due to poor habits of the local population (1, 2). OSCC is usually treated with surgical resection combined with chemoradiotherapy, but the 5-year survival rate for patients with advanced OSCC remains low (3). The tumor microenvironment (TME) comprises immune cells, fibroblasts, blood vessels, and extracellular complexes that are closely associated with OSCC progression (**Figure 1**) (4). Cells in TME can form complex cellular networks by secreting RNAs, proteins, cytokines, and metabolites (5). Moreover, the interactions among cells in TME affect OSCC proliferation, invasion, migration, and angiogenesis (6).

T lymphocytes constitute a crucial component within the immune microenvironment of OSCC. T lymphocytes can be classified into regulatory T-cells (Tregs), T helper (Th) cells, cytotoxic T lymphocytes (CTLs), mucosal-associated invariant T cells (MAIT), natural killer (NK) cells, γδ T cells et al. according to their different surface markers and functions (7). CTLs can control and kill tumor cells to exert an immune effect and Th cells can assist immune cells in removing pathogens (7). NK cells and cluster of differentiation (CD) 8+ T cells possess the capability to recognize and kill tumor cells (8). Tregs foster an immunosuppressive microenvironment, thereby advancing the progression of tumors. It is demonstrated that the downregulation of SLC3A2 regulates immune evasion and promotes metastasis by diminishing the immune capabilities of T lymphocytes in OSCC (9). TME suppresses the immune response by regulating signaling pathways, including VitD signaling pathway, regulating the expression of Tregs and Th cells (10). The functionality of CTLs and Th cells is impeded in OSCC.

The mutations of cancer-related gene, such as tumor suppressor gene *P53* (*TP53*) and HRAS, exert significant influences on head and neck squamous cell carcinoma (HNSCC) as well as the immune mechanisms within TME (11). *TP53* gain-of-function mutation *TP53* decreases CD8+ T cell levels within TME by modulating cytokine expression, which in turn inhibits the infiltration of Tregs and M2 tumor-associated macrophages (TAMs), thereby facilitating immune escape and the progression of OSCC (12). This review summarizes the current literature that describes the role of various cellular components and other TME factors in the progression of OSCC, hoping to provide new ideas for the novel OSCC treatment strategies targeting the complicated cellular network and factors that mediate the interactive loops among cells in TME.

## 2 TAMs

### 2.1 Subtypes and polarization of TAMs

**Figure 2:**
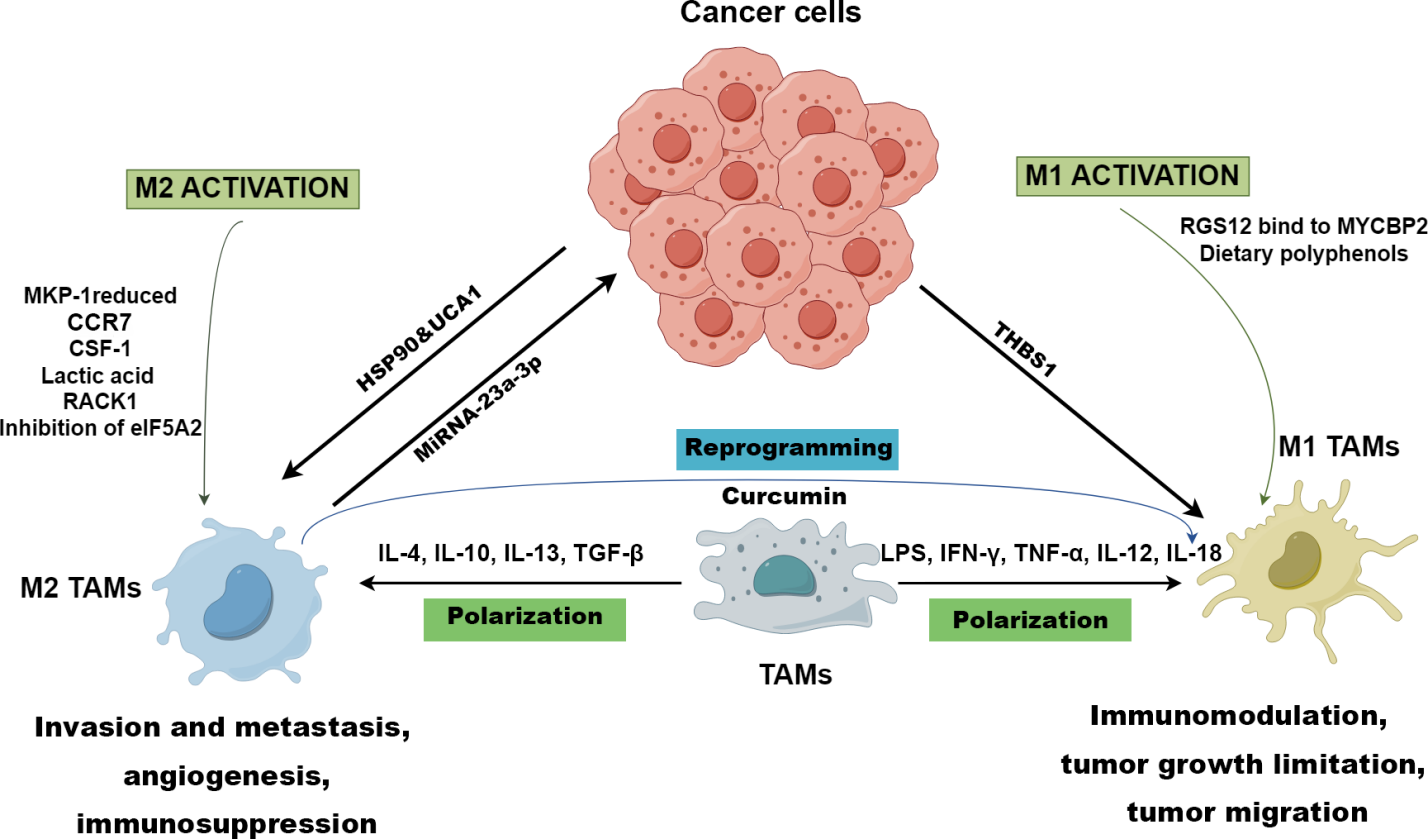
Polarization of TAMs drawn by Figdraw. M1 TAMs polarization is driven by LPS, IFN-γ, TNF-α, IL-12, and IL-18, while IL-4, IL-10, IL-13, and TGF-β promote macrophages towards M2 polarized state. RGS12 binds to MYCBP2 to degrade KIF2A in TAMs driving TAMs to polarize towards the M1 phenotype. However, CCR7, CSF-1, lactic acid, RACK1, reduced MKP-1, and inhibition of eIF5A2 contribute to the genesis of M2 TAMs. M1 TAMs are conducive to immunomodulation, tumor growth limitation, and tumor migration, while M2 TAMs foster invasion and metastasis, angiogenesis, and immunosuppression in OSCC. The TME prompts the polarization of M1 TAMs through cancer-derived exosomal THBS1, while exosomal UCA1 and HSP-90 guide TAMs towards the M2 phenotype polarization. Exosomal miRNA-23a-3p derived from M2 TAMs promotes the proliferation and invasion of OSCC cells and inhibits cancer cell apoptosis. In immunotherapies, and curcumin can reprogram M2 TAMs to M1 TAMs to inhibit OSCC.

Macrophages can be polarized into classically activated macrophages (M1) and alternatively activated macrophages (M2) (**Figure 2**) (13). M1 TAMs polarization is driven by lipopolysaccharides (LPS), interferon-gamma (IFN-γ), tumor necrotic factor alpha (TNF-α), interleukin (IL)-12, and IL-18 (**Figure 2**). M1 TAMs exhibit pro-inflammatory and anti-tumor responses through secreted cytokines, recruitment of cluster of differentiation (CD) 8+ T cells and natural killer (NK) cells, and increased antigen presentation (**Figure 2**). IL-4, IL-10, IL-13, and transforming growth factor beta (TGF-β) promote the polarization of M2 TAMs that displays an anti-inflammatory response and promotes tumor growth, metastasis, and angiogenesis (**Figure 2**) (14, 15).

OSCC cells contributes to the genesis of M2 TAMs through the p38/MKP-1 signaling axis (**Figure 2**) (16). Ono et al. found that heat shock protein 90 (HSP-90)-enriched metastatic oral cancer-derived extracellular vesicles (MEV) can also tune TAMs to M2 phenotype (**Figure 2**) (17). Plasminogen activator inhibitor-1 (PAI-1) and IL-8 derived from OSCC cells have been proved to conduce to the production of CD206+ TAMs (18). Zhou et al. reported that CCR7 in OSCC cells facilitate M2 polarization of macrophages by increasing production of CCL19 and CCL21 (**Figure 2**) (19). Moreover, stromal cells (SCs), verrucous OSCC−associated SCs, and ED−type OSCC-associated SCs are conducive to the differentiation towards M2 phenotype (20). OSCC cells-derived HMGB1and macrophage endogenous HMGB1 facilitate the polarization of M1 macrophages via TLR4/NF-κB signaling (21). Chen et al. demonstrated that OSCC cells-derived THBS1 in exosome promotes M1 macrophages polarization (22). These studies indicate that OSCC cells-derived proteins, enzymes, cytokines and chemokines in TME can induce M2 or M1 polarization of macrophages. However, there are few studies on macrophage polarization induced by other cells besides OSCC cells, and the mechanism has not been fully elucidated. In addition, the studies involved in the regulation of TAMs by HMGB1 secreted during cell death is still controversial.

### 2.2 Role of TAMs in OSCC invasion and migration

#### 2.2.1 M2 TAMs

M2 macrophage-derived exosomal miRNA-23a-3p promotes the proliferation and invasion of OSCC cells and inhibits apoptosis by inhibiting the expression of phosphatase and tensin homolog (PTEN) *in vitro* and *in vivo* (**Figure 2**, **Table 1**) (23). Wang et al. found that TAMs facilitated OSCC migration and invasion via the MIF/NLRP3/IL-1β pathway, which could be disrupted through melatonin (**Table 1**) (24). Increasing colony stimulating factor-1 (CSF-1) levels causes increased infiltration of M2 TAMs, which has an influence on the development and progression of OSCC (**Table 1**; **Figure 2**) (25). Lactic acid-induced M2 TAMs promote OSCC migration and invasion via GPNMB/CD44 pathway (**Figure 2**; **Table 1**) (26). M2 TAMs enhance OSCC migration and metastasis via CCL13 upregulated by Stress Granule (**Table 1**) (27).

**Table 1:** Role of TAMs in OSCC.

Year	Markers	Human/Mouse	Role of TAMs in OSCC	Reference
2023	CD163, CD206	Human	promote proliferation, invasion, and migration of OSCC	Li et al. (23)
2023	CD40, CD163	Human	promote migration and invasion of OSCC	Wang et al. (24)
2020	CD68, CD206	Human	promote progression, invasion, and lymph node metastasis of OSCC	Guo et al. (25)
2023	CD206	Human	promote migration and invasion of OSCC	Lin et al. (26)
2022	CD206	Human	promote migration and metastasis of OSCC	Liu et al. (27)
2024	CD80, CD86	Mouse	promote migration and invasion of OSCC	Jiang et al. (28)
2022	CD68, CD80, CD86	Human	promote EMT and induce CSCs of OSCC	You et al. (29)
2021	CD68	Human	promote invasion of OSCC	Silva et al. (30)
2023	CD68	Human	promote invasion, immunosuppression, and immune escape of OSCC	Wen et al. (21)
2020	CD68, CD163	Human	promote immune escape of OSCC	Suárez-Sánchez et al. (31)
2023	CD163	Human	promote immunosuppression of OSCC	Wang et al. (32)
2022	CD163, CD206, CD11b+	Human	promote immune escape of OSCC	Wu et al. (33)

#### 2.2.2 M1 TAMs

IL-6 derived from High mobility group box 1 (HMGB1)-activated M1 TAMs enhances OSCC migration and invasion (**Table 1**) (28). M1 TAMs polarized by exosomal thrombospondin 1 (THBS1) from OSCC cells can promote the EMT process and induce cancer stem cells (CSCs) through the IL6/Stat3/THBS1 feedback loop (**Figure 2**; **Table 1**) (29).

#### 2.2.3 TAMs

TAMs activation and polarization induced by overexpression of TWIST1 and CSF1 in OSCC cells facilitate OSCC invasion (**Table 1**) (30). TAM-derived HMGB1 promotes OSCC invasion via IL-6/NF-κB/MMP-9 pathway (**Table 1**) (21). Lin et al. found that alpha-enolase (ENO1) and lactic acid derived from tumor cells promotes OSCC migration and invasion by coordinating IL-6 release from TAMs (34).

Although it is widely acknowledged that M2 TAMs promote OSCC invasion and migration through complex signaling pathways involving the secretion of bioactive molecules, M1 TAMs have also been shown to contribute to the progression of OSCC metastasis. The regulation of TAMs polarization and cytokine secretion by lactic acid has gradually attracted the attention of researchers. In particular, TAM-derived IL-6 can significantly promote the invasion and migration of OSCC, which is expected to become a potential therapeutic target. However, the characterization of TAMs phenotype remains inadequate in some studies, and the detailed intracellular signaling pathways related to TAMs polarization and cytokine secretion needs further elucidation.

### 2.3 Role of TAMs in OSCC angiogenesis

M2c and M2d TAMs can elevate glycolysis and initiate angiogenesis with secreted chemokines, fibronectins, and growth factors (35). CSCs, endothelial cells (ECs), and TAMs constitute interrelation through the exchange of cytokine mediators, such as vascular endothelial growth factor (VEGF), IL-6, IL-8, and CSF-1, leading to microvessel density improvement and progression of OSCC (36). ALDH3A1 overexpression in OSCC cells suppresses CRI regulated by TAMs, resulting in inhibition of angiogenesis (37). TAMs play an important role in angiogenesis by producing pro-angiogenic factors. CSCs, ECs, and TAMs construct a sophisticated cellular network, utilizing mediators to regulate angiogenesis in OSCC. Targeting TAMs is an effective way to disrupt the formation of tumor vascular networks and thus inhibit the progression of OSCC. However, the detailed signaling pathways related to the production of pro-angiogenic factors in TAMs and targeted drug interventions need to be further investigated.

### 2.4 Role of TAMs in OSCC immunosuppression

M2 TAMs promote the immune escape of OSCC through upregulating the checkpoint programmed death-ligand 1 (PD-L1) expression (**Table 1**) (31). OSCC cells-derived GM-CSF modulates the level of PD-L1 expression in TAMs via JAK2/STAT3 signaling cascade to promote immune escape (**Table 1**) (32). Endogenous HMGB1 in TAMs promotes immunosuppression and immune escape via the IL-6/STAT3/PD-L1 pathway (**Table 1**) (21). M2 TAMs induced by cancer stem cell-derived *UCA1* targeting *LAMC2* promotes immunosuppression by suppressing CD4^+^ T-cell proliferation and IFN-γ production (**Figure 2**, **Table 1**) (33). A high level of Perilipin 2 (PLIN2) derived from TAMs induces less CD8+ T cells but more TAMs and Foxp3+ Tregs, inducing immunosuppression in OSCC (38). Foxp3+ Tregs contribute to the evasion of immune surveillance through the secretion of cytokines by M2 TAMs in TME (39).

M2 TAMs promote the immunosuppression of OSCC by inducing the expression of PD-L1 and directly inhibiting CD4+ and CD8+ T cells. Inhibition of M2 macrophages polarization can be used as a long-term therapeutic strategy to control or reverse immunosuppression and immune escape. However, the correlation between the density of specific subtypes of TAMs and immune escape remains controversial. The surface receptors and signaling pathways of TAMs that mediate M2 TAMs polarization as well as TAMs-secreted mediators associated with immunosuppression are the focus of further research. Meanwhile, it is necessary to verify whether HMGB1 and PLIN2 can be used as potential therapeutic directions for OSCC.

### 2.5 Immunotherapies targeting TAMs

Currently, immunotherapies targeting TAMs mainly concentrate on three aspects: hindering macrophages from polarizing, removing M2 TAMs from the TME, and repolarizing M2 TAMs towards M1 TAMs (39, 40). Myeloid-based Dusp1-deficiency can reduce the accumulation and polarization of TAMs, which may be a novel target to reprogram TAMs and inhibit the progression of OSCC (41). Targeted inhibition of eukaryotic initiation factor 5A2 (eIF5A2) impedes tumor growth and polarization towards M2 TAMs, which is correlated with the function of modulating cell proliferation, CSC properties, and EMT in oral cancer (**Figure 2**) (42). PY314, an antibody against TREM2, can exhaust TREM2+ TAMs to decrease exhausted CD8+ T cells and the method has already been applied in clinic (43). Regulator of G protein signaling 12 (RGS12) suppresses OSCC by driving M1 TAMs polarization via MYCBP2/KIF2A signaling pathway (**Figure 2**) (44). Dietary polyphenols can promote polarization towards M1 TAMs and curcumin can reprogram M2 TAMs to M1 TAMs to inhibit OSCC (**Figure 2**) (45, 46). Receptor for activated C kinase 1 (RACK1) fosters the polarization towards M2 TAMs improving the M2/M1 TAMs ratio by means of regulating NF-κB, which can be used as a potential therapeutic target (**Figure 2**) (47).

A significant breakthrough has been made in cancer immunotherapy through advancements in nanomedicine. Nanomedicines targeting TAMs plays a very important role in anti-tumor immunotherapy, mainly focusing on inhibiting the recruitment of TAMs, killing TAMs, reprogramming the TAMs phenotype, and promoting the immune function of TAMs (48, 49). Mannose-modified hyaluronic acid nanocapsules targeting TAMs enable precise drug delivery and reprogramming of M2 TAMs (50).

Given the high infiltration of TAMs in OSCC and their intimate involvement in OSCC progression, targeting TAMs has emerged as a promising area of research for therapeutic intervention. However, the diverse subtypes of TAMs and complexity of TME have significantly limited the advance of immunotherapy specifically targeting TAMs in OSCC. Further elucidation of the regulatory mechanism for TAMs polarization and mediator secretion, and the development of TAMs targeting drugs using nanomedicine has become the focus of further research.

## 3 CAFs

### 3.1 Activation of fibroblasts into CAFs

CAFs are activated fibroblasts with mesenchymal cell lineage as well as heterogeneity of phenotype and function in TME (51, 52). CAFs secrete a variety of active factors that are involved in tumor biologic behaviors such as formation and maintenance of tumor mass, generation and maintenance of cancer cell stemness, immune regulation, angiogenesis, metabolic response, ECM remodeling, homeostasis, and treatment resistance (53).

**Figure 3:**
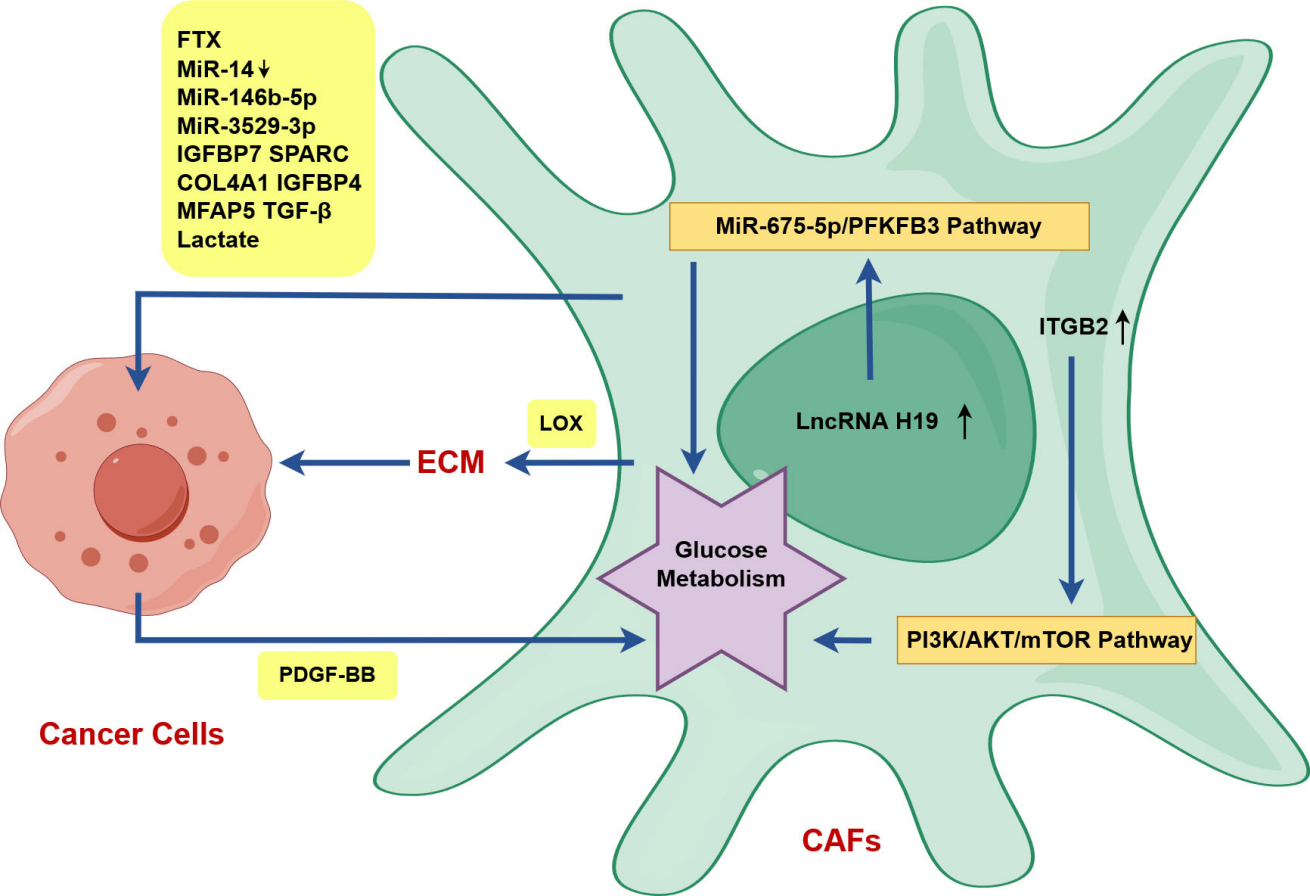
CAFs activate OSCC cells in OSCC drawn by Figdraw. ITGB2 expression in CAFs can improve the glycolysis ability of CAFs by activating the PI3K/AKT/mTOR axis, and upregulated lncRNA H19 in CAFs can promote glucose metabolism via the lncRNA H19/miR-675-5p/PFKFB3 signaling cascade. PDGF-BB-induced glucose metabolism reprogramming in CAFs promotes the proliferation, invasion and metastasis of tongue squamous cell carcinoma (TSCC) via the secretion of lactates. CAFs can directly activate OSCC cells and promote OSCC progression by secreting RNAs, proteins, TGF-β and lactate (FTX, decreased miR-14, miR-146b-5p, miR-3529-3p, IGFBP7, SPARC, COL4A1, IGFBP4, MFAP5, TGF-β, lactate). In particular, CAFs indirectly activate OSCC cells via LOX-mediated the remodeling of stromal collagen microenvironment.

TGF-β activates fibroblasts into CAFs, and lncRNA PVT1 expression further stimulates CAFs proliferation by increasing infiltration of TGF-β (54). Exosomes released from HNSCC cells can convert normal fibroblasts into CAFs (55). Numerous evidences showed that OSCC cells-derived extracellular vesicles (EVs) can activate fibroblasts into CAFs, although EVs vary in content and may include RNAs, proteins and TGFβ (56). CAFs can activate OSCC cells and suppress immune cells, which is related to their secretion of RNAs, proteins, TGF-β and their own regulation of glucose metabolism (**Figure 3**).

### 3.2 CAFs activate OSCC cells

#### 3.2.1 CAFs-derived RNAs activate OSCC cells

The podoplanin (PDPN)+ CAFs secrete exosomal lncRNA FTX to increase the migratory capability of OSCC by suppressing ferroptosis via FTX/FEN1/ACSL4 pathway (**Figure 3**) (57). The overexpression of lncRNA TIRY in CAFs can lead to decreased expression of miR-14 in CAFs-derived exosomes, which facilitates EMT, invasion and metastasis of OSCC cells via Wnt/β-catenin pathway (**Figure 3**) (58). CAFs-derived exosomal miR-146b-5p facilitates OSCC proliferation and metastasis through downregulating homeodomain-interacting protein kinase 3 (HIPK3) expression (**Figure 3**) (59). PDGF-BB-induced CAFs-derived exosomal miR-3529-3p promotes OSCC proliferation, migration, and invasion as well as inhibits apoptosis of cancer cells (**Figure 3**) (60).

#### 3.2.2 CAFs-derived proteins activate OSCC cells

FERM domain containing kindlin 2 (FERMT2) upregulation in CAFs promotes EMT of OSCC by modulating the secretion of insulin-like growth factor binding protein 7 (IGFBP7), secreted protein acidic and rich in cysteine (SPARC), collagen type IV alpha 1 chain (COL4A1), and insulin-like growth factor binding protein 4 (IGFBP4) in oral CAFs (**Figure 3**) (61). CAFs located at the invasive tumor front (CAFs-F), characterized by higher expression of the fibroblast activation marker SMA, were found to secrete elevated levels of microfibril-associated protein 5 (MFAP5) in comparison to NFs and the superficial tumor (CAFs-S) (62). That’s why the degree of EMT and the impact on the invasion and metastasis of OSCC are greater in CAFs-F (62).

Lysyloxidase (LOX) highly expressed in CAFs can remodel the matrix collagen microenvironment, enhance matrix stiffness, and accumulate nuclear β-catenin giving rise to EMT and tumor invasion through the FAK phosphorylation pathway (**Figure 3**) (63). Stromal nicotinamide N-methyltransferase (NNMT), a specific biomarker of CAFs, can regulate type I collagen deposition through epigenetically regulating the transcription of LOX via the NNMT-LOX-FAK axis by reducing the enrichment of H3K27me3, which can foster OSCC stemness by maintaining FAK signaling (**Figure 3**) (64). Mediated by integrin α2β1, CAFs-derived LOX-rich EVs mediate collagen crosslinking through fibronectin, periostin, and bone morphogenetic protein-1, and promote EMT via p-FAK/p-paxillin/YAP signaling pathway (65).

#### 3.2.3 CAFs-derived TGF-β activates OSCC cells

TGF-β can activate OSCC cells via classical SMAD pathway. TGF-β secreted by CAFs in invasive cancer nests could increase the level of sex-determining region Y (SRY)-box9 (SOX9) expression by activating SMAD pathway, which was conducive to OSCC invasion and migration (66). Single-cell analysis showed that CAFs could promote OSCC invasion by secreting TGF-β through SMAD pathway (67).

#### 3.2.4 CAFs-derived lactate activates OSCC cells

Higher integrin beta 2 (ITGB2)-mediated lactate release in CAFs promotes OSCC proliferation by oxidation of NADH in mitochondrial oxidative phosphorylation system (**Figure 3**) (68). LncRNA H19/miR-675-5p/PFKFB3 axis-mediated glycolysis reprogramming in CAFs enhances OSCC growth through lactate secretion (**Figure 3**) (69). PDGF-BB-induced glucose metabolism reprogramming in CAFs promotes the proliferation, invasion and metastasis of tongue squamous cell carcinoma (TSCC) via the secretion of lactates (70).

CAFs can directly activate OSCC cells and promote OSCC progression by secreting RNAs, proteins, TGF-β and lactate. In particular, CAFs indirectly activate OSCC cells via LOX-mediated the remodeling of stromal collagen microenvironment. CAFs-derived exosomal lncRNA and miRNA promote OSCC proliferation, migration, and invasion, but relevant studies on circ RNA are lacking. The mechanism of lactate-related glycolysis reprogramming in CAFs has gradually become a research hotspot. In addition, the mediators secreted by CAFs to activate OSCC cells in some relevant studies have not been clarified, which needs to be further investigated (71).

### 3.3 CAFs suppress immune cells

CAFs-derived exosomal immune-related genes (hsa-miR-139-5p, ACTR2 and EIF6) induce immunosuppression and promote OSCC proliferation by regulating the expression of PIGR, CD81, UACA, and PTTG1IP in cancer cells (72). CAF-derived ACLP suppresses CD8+ T cell migration and facilitates OSCC progression (73). Zhao et al. reported that the low number of CD68+ CAFs, a novel subtype of CAFs, exhibited a negative association with the proportion of Tregs (74).

CAFs can indirectly or directly inhibit immune cells to promote the progression of OSCC. The indirect inhibition effect of CAFs on immune cells is achieved by inducing immunosuppressive microenvironment. Genome sequencing and mRNA-miRNA interaction network analysis provide evidence for the correlation between CAFs-derived exosomal immune-related genes and immunosuppression. However, their detailed regulatory mechanism in OSCC has not been fully clarified due to the lack of comprehensive molecular biological analysis. The next step is to further elucidate the specific mechanism of CAFs inhibiting immune cells through *in vitro* and *in vivo* experiments, while focusing on the regulation and inhibition of CAFs on T lymphocytes.

## 4 T lymphocytes

### 4.1 Role of Tregs in OSCC

Inflammatory cells within TME secrete chemokines like C-X-C motif chemokine (CXCL)12 and CCL17 to recruit Tregs (10, 75, 76). Alternatively, in non-inflammatory conditions, mutations in genes such as epidermal growth factor receptor (EGFR) can modulate signaling pathways, leading tumor cells to produce chemokines like CCL22 that enhance Tregs infiltration (77). Furthermore, the level of Tregs in the TME is also intricately linked to tumor-draining lymph nodes (TDLNs) (78).

Forkhead box P3 (FOXP3) serves as a significant identifier for the presence and function of regulatory T cells (Tregs), which are essential modulators for the immune system (79). Chatzopoulos et al. found that Foxp3+ T cells took part in the immunosuppressive microenvironment in TSCC (80). Hayashi reported that Foxp3+ T cell expression might play a role in nodal metastasis and exhibited a significant correlation with the weak immune response, onset of OSCC, and enhancement of tumor malignancy (81). However, Koike et al. reported that Foxp3+ T cells might not suppress immune response but might perform site-specific anti-tumor response in OSCC (82). The Foxp3+ T cell density at the invasive front exhibits a positive correlation with overall survival (OS), disease-specific survival (DFS), recurrence-free survival, and metastasis-free survival, which means Foxp3+ T cells may be a novel prognostic factor for OSCC (82). IL-23R- Tregs display a more immunosuppressive profile compared to IL-23R+ Tregs, which is regulated by IL-23 secreted by other cells in TME (83).

### 4.2 Role of CTLs in OSCC

**Figure 4:**
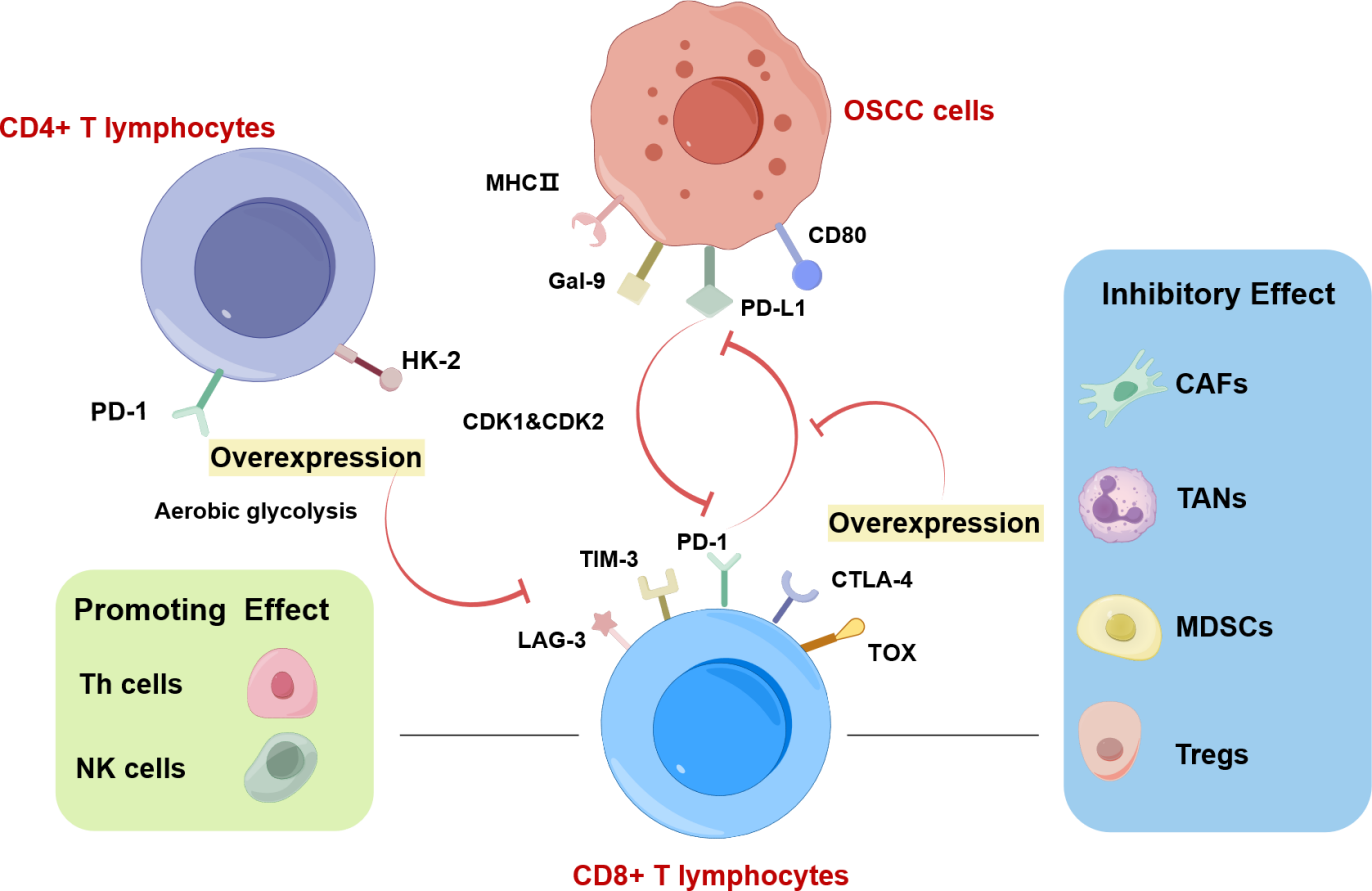
CD8+ T lymphocytes in OSCC drawn by Figdraw. Increased PD-1 levels and glycolysis in CD4+ T cells are positively associated with lymph node metastasis of OSCC. The interaction between CD8+ T cells and cancer cells is regulated by immune cells in TME. Among them, CAFs, TANs, MDSCs, and Tregs exhibit inhibitory effects on CD8+ T lymphocytes. Conversely, NK cells and Th cells play a supportive role in promoting CD8+ T lymphocyte function. The highly expressed T lymphocyte proliferation regulator-related genes (RANs) (CDK1 and CDK2) in malignant cells and T cells, can inhibit the function of CD8+ T cells. CD8+ T cells overexpressing PD-1 or TOX in OSCC have low cytotoxicity and weak ability to proliferate, which is associated with aerobic glycolysis. Exhausted CD8+ T cells are characterized by upregulated immunosuppressive checkpoints including PD-1, TIM-3, and CTLA-4.

The expansion of CD4+ cytotoxic T lymphocytes with specific gene expression profiles may inhibit the tumor immunity in OSCC (84). Increased PD-1 levels and glycolysis in CD4+ T cells are positively associated with lymph node metastasis of OSCC (**Figure 4**) (85). Statistical analysis revealed that the level of CD8+ T cells in TSCC exhibited a crucial correlation with the tumor inflammation signature and cytotoxicity signature (80). CD8+ T cells with the overexpression of PD-1 or TOX in OSCC have low cytotoxicity and weak ability to proliferate, which impairs the function of immunosuppression (**Figure 4**) (79). Exhausted T cells are characterized by upregulated immunosuppressive checkpoints including PD-1, TIM-3, and CTLA-4 (**Figure 4**) (86). Exhausted CD4+ T cells which appear earlier and more in number than exhausted CD8+ T cells foster the development of oral cancer (86).

### 4.3 Role of other T lymphocytes in OSCC

A high ratio of CD45RO-expressing tumor-infiltrating lymphocytes (TILs) was associated with a disease-free/overall survival improvement in OSCC patients, suggesting that CD45RO-expressing TILs are useful biomarkers for OSCC (87). Caspase activation in TILs is associated with lymph node metastasis in OSCC (88). malignant cells and T cells overexpress T lymphocyte proliferation regulator-related genes (RANs) to maintain the immunosuppressive microenvironment by impeding the functionality of plasmacytoid dendritic cells (pDCs) and CD8+ T cells but enhancing the activity of Th2 cells and Th cells (**Figure 4**) (89).

In conclusion, Tregs exhibit immunosuppressive properties, yet there are also reports indicating their anti-tumor effects. CTLs possess the capability to eliminate tumor cells, thereby inhibiting OSCC and being linked to a positive prognosis. The role of Tregs in OSCC is intricate, and their impact on the prognosis of OSCC patients remains contentious. Given this complexity, there is a pressing need for more comprehensive and in-depth research into Tregs.

## 5 TANs

Tumor-associated neutrophils (TANs) are a kind of tumor-associated myeloid cells derived from blood neutrophils in TME (90). Due to their plasticity, TANs can polarize into distinct phenotypes upon stimulation by various cytokines and growth factors, which can be broadly categorized as N1 TANs and N2 TANs (91, 92). N1 TANs are related to the process against tumor proliferation with direct cytotoxicity, while N2 TANs can promote tumor progression (91). The overexpression of chemerin, a kind of effective chemoattractant protein derived from tumor cells, can improve the number of TANs in TME (93). TANs-derived small extracellular vesicles (sEVs) can also promote as well as inhibit tumor growth (94).

The pro-tumor functions of TANs include the promotion of proliferation and anti-apoptosis, EMT, invasion, metastasis, nodal spread, tumor vascularization, and immunosuppression (91, 95). B-cell activating factor (BAFF) secreted by TANs promotes proliferation of OSCC cells and hinders their apoptosis (96). TANs contribute to OSCC proliferation, invasion, and migration via JAK2/STAT3 signaling pathway and EMT with the help of chemerin (93). Lonardi et al. revealed that a high proportion of CD66^+^ TANs in TDLNs had been found in OSCC patients with worse prognosis which indicated TANs might promote nodal spread through lymphatic vessels (97). TANs also play a role in OSCC through the network with other myeloid immune cells to affect prognosis (98). The upregulation of ALDH3A1 serves to impede the polarization of neutrophils towards the N2 phenotype and also hinders their migration by inhibiting IL-8 secretion to deactivate the PI3K/AKT/NF-κB pathway, which is conducive to inhibiting inflammation and halting oral cancer growth (99).

Interactions between OSCC cells and TANs modulate the behavior of TANs and reprogram them to mediate pro-inflammatory or immunosuppressive responses. OSCC cells- and TANs-derived sEVs carry complex mediators which finally are delivered to respective recipient cells in TME, constructing the complete positive feedback interactive loops. Although the details of complex bidirectional crosstalk remain unknown, a better understanding of the interactive loops might provide new treatment strategies for OSCC in the future.

## 6 MDSCs

Hematopoietic precursor cells in bone marrow make up MDSCs, which can be classified into polymorphonuclear MDSCs (PMN-MDSCs) and monocytic MDSCs (M-MDSCs) (100, 101). OSCC cells can facilitate the process of transforming to MDSCs with immunosuppressive phenotype (102). OSCC-derived MDSCs can foster the proliferation, migration, and poor prognosis of cancer cells (102). MDSCs can also induce EMT in OSCC cells, which is conducive to vasculogenic mimicry formation (102). Kouketsu et al. reported that MDSCs had an inhibitory effect on T cells leading to the reconstitution of TME, which was associated with the oncogenesis of OSCC (103). Moreover, Jiang et al. reported that the elevated proportion of CD11b+ MDSCs could promote the generation of tumors (104). The presence of CD11b+ MDSCs in both the core and invasive margins of OSCC exhibits a strong association with the lower OS and DFS of OSCC, indicating their potential as a prognostic factor (104). At present, most studies on the role of MDSCs in OSCC remain in the correlation aspect, and there are few studies involved in the detailed mediators, receptors and intracellular signaling pathways. Further studies should focus on the molecular mechanism of MDSCs regulation in OSCC, which is helpful to provide potential targets for intervention.

## 7 Cytokines

### 7.1 Role of TNF in OSCC

TNF-α and IL-8 are prominently overexpressed in OSCC and OPMDs, significantly contributing to the advancement of malignant transformation (**Table 2**) (105). Gupta et al. reported that both TNF-α and TNF-β took an active part in the progression of precancerous lesions and OSCC in the North India population, but the influence of TNF-α was stronger (**Table 2**) (106). OSCC patients have abundant rare *in situ* cytokine-secreting T cell populations such as CD4+ Th cells secreting TNF-α and CD8+ T cells secreting IFN-γ (**Table 2**) (107). The antigen-reactive memory T cells promote tumor immune control through the secreted type I cytokines, especially TNF-α (**Table 2**) (107).

**Table 2:** Role of cytokines in OSCC.

Cytokines	Role of Cytokines in OSCC	Mechanism	Reference
TNF-α	promote advancement of malignant transformation	NA	(105–107)
	promote progression	NA	(106)
	promote tumor immune control	NA	(107)
TNF-β	promote progression	NA	(106)
TGF-α	promote proliferation and metastasis	by suppressing immune responses and promoting angiogenesis	(108)
TGF-β	promote EMT and metastasis	by promoting EndMT	(109)
		by promoting the polarization towards M2 TAMs	(110)
IL-6	promote EMT and migration	through the interaction of IL-6-ATATT3 axis and Rac1 axis	(113)
	change TME	by recruiting inflammatory cells and reducing the production of TIMP-1	(114)
	promote proliferation	by up-regulating anti-apoptotic genes and promoting cell proliferation via a STAT-dependent pathway	(112)
IL-8	promote advancement of malignant transformation	NA	(105, 114)
	change TME	by recruiting inflammatory cells and reducing the production of TIMP-1	(114)
IL-17	promote proliferation and metastasis	by activating the IL-17/TRAF6/AP-1 signaling pathway	(115)
VEGF	promote angiogenesis and distant metastasis	through the VEGF/VEGFR pathway	(116)

### 7.2 Role of TGF in OSCC

TGF-α has been linked to facilitating cell proliferation, and metastasis, suppressing immune responses, and promoting angiogenesis (**Table 2**) (108). TNF-α promotes TGF-β-induced endothelial-to-mesenchymal transition (EndMT) via TGF-β signal augmentation in OSCC (**Table 2**) (109). Furthermore, Maldonado et al. demonstrated that suppressing the secretion of TGF-β in TME would interfere with the polarization of M2 TAMs, inhibiting invasion and chemotaxis of OSCC cells (**Table 2**) (110).

### 7.3 Role of interleukin in OSCC

Lopez-Labady et al. have reported that the level of IL-1β and IL-8 in OSCC was comparatively less than that found in normal mucosal tissue, indicating that the chemotactic activity and anti-tumor function are decreased in OSCC (111). IL-6 can contribute to tumor growth by up-regulating anti-apoptotic genes and promoting cell proliferation via a STAT-dependent pathway (**Table 2**) (112). IL-6 can facilitate the migration ability of OSCC cells undergoing EMT through the interaction of IL-6-ATATT3 axis and Rac1 axis (**Table 2**) (113). IL-6 and IL-8 may alter TME by recruiting inflammatory cells and reducing TIMP-1 production, and may serve as potential prognostic biomarkers for OSCC (**Table 2**) (114). OSCC-derived EVs can promote tumor proliferation and invasion by altering the levels of inflammatory cytokines in TME, and activating the IL-17/TRAF6/AP-1 signaling pathway (**Table 2**) (115).

### 7.4 Role of VEGF in OSCC

In OSCC, overexpressed VEGF participates in angiogenesis through the VEGF/VEGFR pathway, which is related to the occurrence of lymph node and distant metastasis and poor prognosis of OSCC (**Table 2**) (116).

Cytokines, including IL-8, TNF-α, TNF-β, CXCL9, CXCL10, CCL5, CCL17, CCL22, and IL-10, exhibit close correlations with the progression of OPMDs to OSCC by fostering inflammation, angiogenesis, and immune modulation. Similarly, TGF, IL, TNF, and VEGF play pivotal roles in OSCC development by stimulating the proliferation and migration of OSCC cells, modulating the immune response and angiogenesis, and influencing bone resorption. As a result, these cytokines hold promise as potential novel prognostic biomarkers for clinical diagnosis and treatment of OSCC. However, the precise role of various cytokines in oral squamous cell carcinoma (OSCC) remains incompletely understood. Therefore, future research is necessary to delve deeper into the specific mechanisms and effects of these cytokines in OSCC, with the aim of identifying potential targets for immunotherapy.

## 8 Hypoxia

### 8.1 Role of hypoxia in OSCC

Hypoxia biomarkers exhibit an association with early dysplastic epithelial changes, which indicates that hypoxia has a potential effect on oral carcinogenesis (117). Hypoxia exhibits a significant association with immune cell infiltration and gene mutations in OSCC (118). Hypoxic TME induces changes to histone acetylation and methylation in OSCC (119). Under hypoxia, the upregulated Tumor protein D52 (TPD52) expression at the center of OSCC is beneficial to cell viability and proliferation by inhibiting autophagic signal transduction (120). Overexpression of special AT-rich sequence-binding protein 2 (SATB2) in SCC9 cells induced by hypoxia increases the stemness of SCC9 cells and accelerates autophagy by affecting the level of Beclin-1 and the conversion of LC3-I (121). The level of regulated in development and DNA damage responses 1 (REDD1) that induces hypoxia considerably increases in OSCC leading to rising microvessel density and lymphatic metastasis (122). Suprabasin elevates the invasion, migration, and angiogenic ability of OSCC in hypoxia (123). Carbonic anhydrase 9 (CA9) is frequently expressed in TSCC due to hypoxia, which can regulate oncogenes and signaling pathways such as apoptosis, hypoxia, PI3K/AKR/mTOR signaling (124). The expression of Phosphoglycerate kinase 1 (PGK1) is also increased because of hypoxia, which engages the AKT signaling pathway to foster glycolysis, facilitate cell growth, and contribute to metastasis by promoting EMT (125). Vacuolar ATPase (V-ATPase), an enzyme that influences cellular glycolysis, primarily regulates potential of hydrogen (pH) within TME, and autophagy triggered by V-ATPase may contribute to the development of chemotherapy resistance in OSCC (126). Additionally, the inhibitory effect of odecylmethylaminoethyl methacrylate (DMAEM) on OSCC is enhanced by the low pH in hypoxia conditions (127).

### 8.2 Role of HIF-1α in OSCC

The positive correlation among the expression of CD44, HIF1α, and Snail in advancing grades of OSCC and their metastatic lymph suggests that there is a dynamic interaction between CSCs, hypoxia, and EMT, which facilitates the formation and maintenance of oral CSCs (128). Bharti et al. found the overexpression of HIF-1α in T1-staged OSCC (129). HIF-1α exhibits a crucial correlation with the deterioration of precancerous lesions and the poor prognosis of OSCC (130). Swartz et al. demonstrated the level of HIF-1α expression in OSCC cells is notably higher than that in laryngeal squamous cell carcinoma (LSCC) and oropharyngeal squamous cell carcinoma (OPSCC) (131). They found that HIF-1α had a positive correlation with better OS and DFS which is different from the finding in LSCC and OPSCC (131). There exists an important statistical correlation between the overexpression of HIF-1α and enlargement in tumor size, which means HIF-1α has a potential diagnostic role and maybe a novel auxiliary diagnostic indicator (132).

Hypoxia improves the sensitivity of HSC-2 to lactoferrin (LF) which is an iron-binding glycoprotein causing HIF-1α, HIF-2α, and *p53* protein expression upregulated (133). Expression of HIF-1α can modulate the level of Par3 and TJs expression in OSCC, and the crosstalk between partitioning-defective protein 3 (Par3) and tight junctions (TJs) is disrupted during hypoxia, facilitating the metastasis of OSCC by upregulating MMP activity (134). The PER1/HIF-1α negative feedback loop facilitates ferroptosis and suppresses OSCC progression (135). MicroRNA-18a performs a detrimental function in the migration and invasive capabilities of OSCC cells by downregulating the level of HIF-1α (136). HIF-1α serves as a crucial hypoxia indicator that impacts the prognosis of OSCC by regulating TME and the function of OSCC cells. However, the influence of HIF-1α on prognosis remains controversial, necessitating an investigation into the pathway through which HIF-1α operates within the TME, ultimately targeting HIF-1α as a pivotal therapeutic focus.

### 8.3 Role of hypoxia-relative genes in OSCC

Lan et al. reported that hypoxia-relative genes contributed to the increase of CD68+/CD163+ TAMs and Albumin Human Serum-coated perfluorocarbon-carrying oxygen was an effective way to alleviate hypoxia (137). Hypoxia-related genes, aldolase A (ALDOA), prolyl 4-hydroxylase subunit alpha-1 (P4HA1), PGK1, and VEGFA, exhibit a significant correlation with OS in OSCC, which can predict the prognosis of patients (138). Lu and Liu found a positive correlation between phosphofructokinase, platelet (PFKP) and serpin peptidase inhibitor, clade E, member 1 (SERPINE1), which could also lead to poor prognosis (139). Furthermore, Han et al. revealed that the mutation of nuclear receptor binding SET domain protein 1 (NSD1) also indicated a high hypoxia potential index in OSCC (118). Hypoxia leads to elevated levels of miR-1825 in OSCC cells, promoting angiogenesis via the exosomal miR-1825/TSC2/mTOR signaling pathway (140). Piwi-interacting RNA-39980 inhibits the proliferation and migration of TSCC by suppressing the oncogene farnesyl-diphosphate farnesyltransferase 1 (FDFT1), disrupting ROS production, fostering a hypoxic TME, elevating levels of damaged DNA, and ultimately triggering cell death (141). In hypoxic conditions, hsa_circRNA_101036 boosts endoplasmic reticulum (ER) stress via the miR-21-3p/transmembrane and tetratricopeptide repeat containing 1 (TMTC1) pathway, ultimately causing cell apoptosis and generating ROS that can be removed by peroxiredoxin 5 (142, 143).

In hypoxic TME, hypoxia-relative genes facilitate cell death by modulating ROS levels, resulting in the inhibition the proliferation and migration. Meanwhile, Hypoxia promoting angiogenesis via the increased exosomal miR-1825. Conversely, the upregulation of numerous hypoxia-relative genes leads to an increase in microvascular density or enhances the serum’s oxygen-carrying capacity, thereby providing adequate nourishment and oxygen to OSCC.

## 9 Conclusion

TME has a significant impact on the regulation of OSCC initiation, development, and progression. Various cells in TME form an immunosuppressive environment to promote OSCC angiogenesis, proliferation, invasion, and migration through the cellular network with excretive secreting RNAs, proteins, cytokines, and metabolites. Therefore, it is pivotal to explore the interaction between cancer cells and the immune system in TME for achieving effective treatment of OSCC.A profound understanding of the crosstalk between tumor cells and various cell types in TME may allow the development of novel treatment strategies to relieve immunosuppression in OSCC. It has been reported that numerous immunotherapies targeting TME have been investigated for colon, breast, gastric, and lung cancer, while few have looked at therapies relevant to OSCC. Thus, more investigation needs to be advanced to offer new perspectives on treatment strategies targeting TME, including the complicated cellular network and factors that mediate the dynamic loop.
